# A Novel Four-Step Algorithm for Detecting a Single Circle in Complex Images

**DOI:** 10.3390/s23229030

**Published:** 2023-11-07

**Authors:** Jianan Cao, Yue Gao, Chuanyang Wang

**Affiliations:** School of Mechanical and Electrical Engineering, Soochow University, Suzhou 215137, China; 20215229041@stu.suda.edu.cn (J.C.); cywang@suda.edu.cn (C.W.)

**Keywords:** single-circle detection, Canny edge detection, clustering algorithm, improved least squares method

## Abstract

Single-circle detection is vital in industrial automation, intelligent navigation, and structural health monitoring. In these fields, the circle is usually present in images with complex textures, multiple contours, and mass noise. However, commonly used circle-detection methods, including random sample consensus, random Hough transform, and the least squares method, lead to low detection accuracy, low efficiency, and poor stability in circle detection. To improve the accuracy, efficiency, and stability of circle detection, this paper proposes a single-circle detection algorithm by combining Canny edge detection, a clustering algorithm, and the improved least squares method. To verify the superiority of the algorithm, the performance of the algorithm is compared using the self-captured image samples and the GH dataset. The proposed algorithm detects the circle with an average error of two pixels and has a higher detection accuracy, efficiency, and stability than random sample consensus and random Hough transform.

## 1. Introduction

### 1.1. Background

Single-circle detection has many application scenarios, namely for use in automated inspection and assembly, the identification of weld joints and weld seams, PCB hole detection, and non-destructive testing [1,2,3,4,5]. For example, only the single circle in the image needs to be detected when welding the inner diameter edges of tube heat exchanger bores utilizing machine vision. In the abovementioned fields, the circles to be detected are usually present in complex images. Complex images are images with multiple contours, intricate textures, mass noise, and various levels of brightness, generally containing a lot of information about edge and structure. Therefore, the task of circle detection in complex images and obtaining the localization and shaping parameters of the circle is more challenging. Widely used circle parameter detection methods include random sample consensus, stochastic Hough transform, and least squares, with good robustness and accuracy [6,7].

### 1.2. Literature Review

Hough transform (HT) has received attention from a wide range of scholars due to its insensitivity to noise and ease of implementation in parallel computing. However, the HT algorithm has a long computation time and requires a large storage space, making circle detection inefficient. To solve this problem, Xu et al. [8] proposed the randomized Hough transform (RHT). The RHT algorithm maps multiple pixels on the edge to a single point in the parameter space. It determines the circle’s parameters by randomly obtaining three points in the parameter space, which can significantly reduce the computation time and storage space. Consequently, many scholars have conducted studies based on the RHT algorithm [9,10]. Nonetheless, the algorithm uses three points instead of all points along the circle edge in order to determine the circle parameters, which may reduce the detection accuracy. To enhance the probability that the three points belong to the same circle, Wang [11] proposed an improved RHT method integrated with a fitting subpixel circle detection algorithm, where he removed isolated points after edge extraction. The method effectively eliminates the noise and improves circle detection accuracy. To better remove noise and determine the fitted sample points, Jiang [12] proposed an efficient stochastic Hough transform circle detection algorithm based on probabilistic sampling and feature points, which optimizes the methods of determining sample points and finding candidate circles. Experimental results show that the algorithm improves the effectiveness of sampling the fitted sample points and prevents the fake circles from being regarded as candidate circles. Both the RHT algorithm and the improved algorithm based on RHT obtain the circle parameters via random sampling, which is challenging to apply to circle detection in complex images because the number of noise points in complex images is more than the number of feature points at the circle edge.

Randomized sample consensus (RANSAC) was proposed by Fishler and Bolles in 1981 as an iterative uncertainty algorithm for estimating parameters from noise datasets; this has been used in various image processing and computer vision applications [13]. When fitting a circle, a part of the sample points is randomly selected as the set of fitted sample points, and a circle is fitted. Kiddee et al. [14] used the RANSAC algorithm to determine the location of the edge feature points in circular weld tracking. Although the algorithm can estimate the parameters of the circle edges, it is only suitable for cases where there are fewer noise points outside of the circle. To solve the problem of excessive noise points outside the circle, Ma et al. [15] proposed a spatial circle center fitting method based on the RANSAC algorithm, which reduces the noise outside the circle and improves the robustness of the circle detection. However, circle detection in complex images is still unable to obtain an excellent fitting effect.

Least squares fitting of circles (LSC) [16,17,18] is the method of fitting a circle by minimizing the sum of the squares of the distances between the sample points and the corresponding points on the fitted circle, which has a high fitting accuracy and faster detection speed compared with the RHT and RANSAC algorithms. However, the results obtained based on the LSC algorithm are easily affected by noise. Therefore, scholars have improved and compared the LSC algorithm. Zhou et al. [19] proposed the MFLS algorithm, which removes the noise points by establishing a mathematical model in polar coordinates and then uses the LSC algorithm for circle fitting. The method has high positioning accuracy. However, the detection results may be seriously affected when the noise points are not entirely removed. To detect the parameters of the punched circle quickly and accurately, Cao et al. [20] proposed a circle fitting method based on LSC and the mean shift algorithm. This algorithm concentrates the center of the fitted circle around the true circle’s center in order to obtain the best actual circle. Experiments show that this algorithm detects circles faster than the RHT algorithm. 

In addition to the methods mentioned above, AI-based approaches, e.g., deep learning, have also been used in the literature to detect circle contours. Essentially, AI-based circular contour detection methods usually have high accuracy and robustness. However, their performance depends on the algorithms used and the quality of the training data. The recognition results are usually good if the algorithms and models are adequately trained and have good generalization capabilities. However, some false or missed detections may occur for complex or noisy image scenes.

In summary, the increased complexity of images in which the target circles are located leads to some limitations of the detection algorithms. RHT and RANSAC algorithms are designed based on the random sampling method to obtain the fitted circle. They fit the circle by selecting some of the sample points (pixels at the edges) instead of the sample points of the whole circle, which may lead to the selection of sample points that are not representative enough, especially when the sample points contain noise or outliers. The LSC algorithm has high accuracy but is extremely sensitive to noise. In addition, excessive sample points increase the complexity of the least squares’ nonlinear optimization computation. Thus, a new single-circle detection algorithm is desired for its application in complex images. 

### 1.3. Organization

The rest of the paper is organized as follows: Section 2 states the single-circle detection problem, Section 3 describes the proposed single-circle detection principle, Section 4 performs comparative experiments and results analysis, and Section 5 concludes the paper and discusses the future work.

## 2. Problem Statement

Although single-circle detection with a simple background is a typical computer vision problem, which has been well solved in the literature, single-circle detection with a complex environment requires a more efficient and accurate method. The expected detection method addresses the following four issues:

- The removal of mass noise in the image edge preprocessing stage. Interfering points are an adverse factor affecting the accuracy and efficiency of single-circle detection. Mass noise increases the difficulty of de-noising and main contour detection; therefore, the noise needs to be removed accurately when detecting single circle with a complex background.
- The selection of sample points for fitting circles. After image edge processing, interfering points affect the fitting results. These interfering points are scattered in a low-density region. In contrast, the sample points of the main contour are connected in an arc and are more tightly connected in a high-density area. Considering the characteristics of the interfering points and sample points, establishing a sample point selection method for fitting candidate circles is another challenge.
- The iteration of candidate circles and determination of ideal circles. Overfitting and underfitting are prevented via suitable methods during the exact fitting of circles. We need to find an effective and fast iterative solution for the candidate circle, which in turn ensures the quality of the ideal circle.
- The improvement of output circle detection accuracy. Despite reducing the frequency of overfitting and underfitting occurrences, there may still be an error between the ideal circle and the real-world circle due to the influence of various interfering points. To improve the accuracy of output circle detection, the effect of interfering points on output circle parameters needs to be further reduced.

Definitions of terms used in the methods are given below.

**Definition 1.**  *Candidate circle denotes the circle constructed by fitting during the process in the least squares fitting circles’ iteration process. It cannot be directly output as the final result, and needs further analysis and screening*.

**Definition 2.**  *Ideal circle denotes the last circle fitted by the least square method in Section 3.3*.

**Definition 3.**  *Output circle denotes the final circle output, which is expected to be with high accuracy and stability*.

**Definition 4.**  *Edge detection denotes a method to extract the edges of an image with a large gradient, which includes the circle edges to be detected and the interference points*.

**Definition 5.**  *Main contour denotes the target edge to be detected in the image*.

**Definition 6.**  *Sample points denote pixels in an image. In this paper, the corresponding pixels are put into the coordinate system to explain the principle of each algorithm. Therefore, the pixels are called sample points*.

**Definition 7.**  *Data points in K-means algorithm represent the coordinate values of the center and radius of all candidate circles*.

## 3. Methods

**Figure 1:**
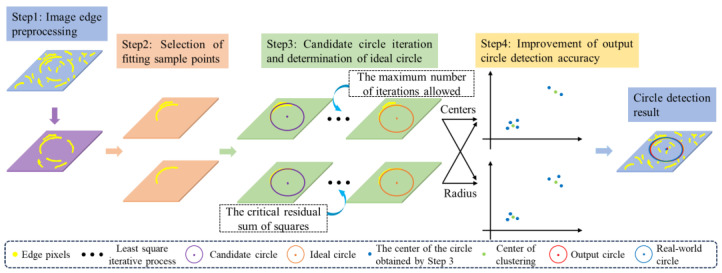
Single-circle detection process.

The algorithm is proposed for single-circle detection, which combines the DBSACN clustering algorithm, the least squares method (LS), and the K-means clustering algorithm. To briefly express our proposed algorithm, it is named as DBLSKCF algorithm. The four steps in the DBLSKCF algorithm respond to the abovementioned four challenges. The complete single-circle detection process is schematically shown in Figure 1.

- Step 1. Image edge preprocessing.
- Step 2. Selection of sample points for fitting curves.
- Step 3. Iteration of candidate circles and determination of the ideal circles.
- Step 4. Accuracy improvement of the output circle detection.

### 3.1. Image Edge Preprocessing

Image edge preprocessing is the foundation for extracting target edges and aims to highlight real and valuable information. However, the images often contain noise due to the impact of uncertainties, such as acquisition equipment and lighting conditions. Therefore, image edge processing, which consists of two key steps, Canny edge detection and main contour screening, can significantly improve single-circle detection performance.

#### 3.1.1. Canny Edge Detection

Edge detection is used in many object edge detection applications to observe image features based on a significant change in the gray level. In addition, it can reduce the amount of data in an image while preserving its structural properties [21]. Therefore, the classical Canny edge detection algorithm is used for the extraction of edge features in images [22]. The edge detection accuracy depends on the thresholds, and a series of pre-experiments are conducted to determine the appropriate thresholds. By analyzing the pre-experimental results, applicable high and low thresholds are selected to extract information about the target edges.

Differentiation is the basis of gradient computation, which is very sensitive to the image’s mutations (generally denotes noise). To improve the accuracy of the detection results, the image needs to be filtered before edge detection to remove interfering points and reduce pseudo edges. Gaussian filtering is effective in smoothing the image and reducing distracting points.

The Gaussian kernel size and the standard deviation affect the filtering effect. The standard deviation in this algorithm uses the default parameter. To determine the optimal Gaussian kernel size, this section performs the filtering process by filtering three complex images with Gaussian kernel sizes of 5 × 5, 7 × 7, 9 × 9, 11 × 11, respectively. The filtered images are then subjected to edge detection using the Canny edge detection algorithm. The results are as follows.

**Figure 2:**
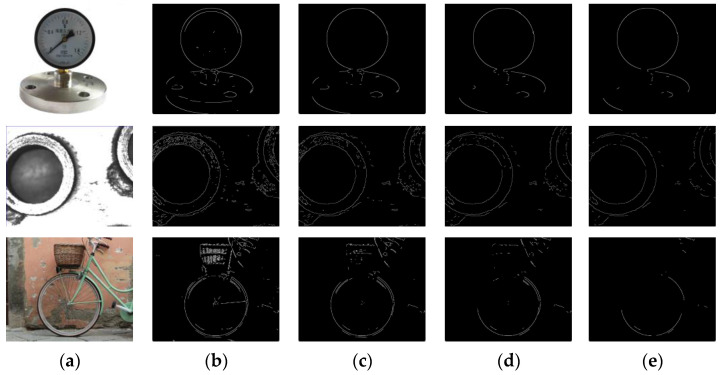
Filtering effect of Gaussian kernels of different sizes. (**a**) Original image; (**b**) 5 × 5; (**c**) 7 × 7; (**d**) 9 × 9; (**e**) 11 × 11.

As shown in Figure 2, the Gaussian kernel size is 5 × 5, 7 × 7, and the corresponding edge detection results have many interfering points and pseudo edges around the detected edges. This phenomenon may reduce the single-circle detection accuracy. The filtering effect is better when the Gaussian kernel size is 9 × 9, 11 × 11. Nevertheless, excessive Gaussian kernel size may cause some target edges to be filtered out, which leads to significant deviations in the circle detection results. Thus, the Gaussian kernel size chosen in the DBLSKCF algorithm is 9 × 9.

#### 3.1.2. Main Contour Screening

Canny edge detection results show that the edges of the main contour are more tightly connected. Meanwhile, the interfering points are mostly irregularly distributed, making it difficult to form a complete edge. Even if these interfering points are connected to form an edge, the length of the edge will be much smaller than the length of the main contour. Based on this feature, the edge lengths are utilized to achieve the main contour screening. Precisely, we can calculate the length of each edge and arrange these lengths in descending order to obtain a sequence of edge lengths. We select a few longer edges to narrow the main contour range and determine the number of retained edges by setting a threshold. The calculation formula is as follows:

(1) $$C_{m}=\left\{e_{1},e_{2},\cdots ,e_{m}\right\}$$

(2) $$C_{s}^{0}=\left\{e_{1}^{0},e_{2}^{0},\cdots ,e_{s}^{0}\right\}(s\leq m)$$

(3) $$C_{p^{s}}=\left\{p_{1}^{s},p_{2}^{s},\cdots ,p_{l}^{s}\right\}$$

where $C_{m}$ denotes the set of all edges before sorting, $e_{1},e_{2},\cdots ,e_{m}$ represent the edge in the image, $C_{s}^{0}$ denotes the set of the first s long edges after sorting, $e_{1}^{0},e_{2}^{0},\cdots ,e_{s}^{0}$ denote the first s long edges after sorting, $C_{p^{s}}$ denotes the set of pixels of the edges in $C_{s}^{0}$, and $p_{1}^{s},p_{2}^{s},\cdots ,p_{l}^{s}$ denote the pixel at the edge in $C_{p^{s}}$. Please note that $C_{p^{s}}$ is the pixel set output after the step of image edge preprocessing.

**Figure 3:**
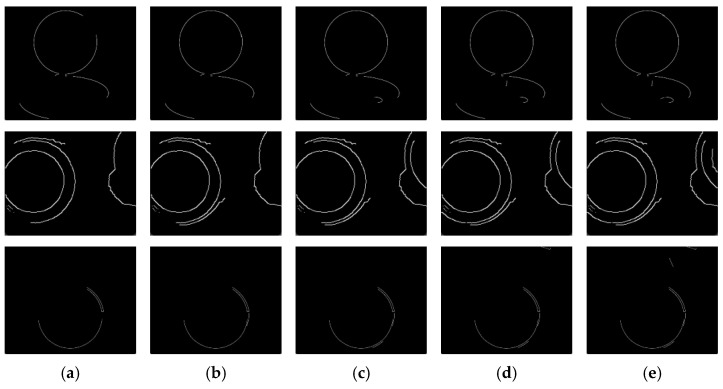
Retention results for different numbers of edges: (**a**) 4, (**b**) 5, (**c**) 6, (**d**) 7, (**e**) 8.

The threshold directly affects the accuracy and efficiency of single-circle detection. In the Canny edge detection algorithm, some irrelevant small edges may be detected due to noise, influencing the circle detection process. The DBLSKCF algorithm keeps a few edges with longer lengths to improve the accuracy and efficiency of circle detection. The length of the edges can be used to assess their continuity. Usually, longer edges are more representative of a part of the real-world circle. To determine the optimal number of retained edge, 4–8 long edges are kept for each of the images in Figure 2d, and the results are shown in Figure 3.

To better express the significance of retaining different numbers of edges, the denoising ratio is introduced in this paper. As shown in Formula (4), the denoising rate indicates the ratio of the number of removed interfering points to the number of detected edge pixels, revealing the denoising ability of the image. The algorithm’s performance under different interfering points levels can be evaluated by retaining the comparison experiments with distinct edges. The computational expression is as follows:

(4) $$\beta =\frac{N_{m}-N_{p^{s}}}{N_{m}}$$

where $N_{m}$ means the number of pixels after edge detection. $N_{p^{s}}$ indicates the number of pixels in the set $C_{p^{s}}$ and β represents the denoising rate.

Main contour screening aims to select the fitted sample points better. If the denoising rate is excessively low, it will lead to many interfering points in the fitted samples, which may reduce the accuracy and efficiency of the fitting. On the contrary, although raising the denoising rate reduces the number of interfering points, it may result in a lack of representative contour points in the fitted sample, adversely affecting the accuracy of the detecting results. The DBLSKCF algorithm integrates the final detection results while increasing denoising to ensure detection efficiency. Additionally, it chooses to retain six edges to obtain accurate fitting results. This strategy enables the DBLSKCF algorithm to obtain better results in real-world circle detection. 

Image edge preprocessing improves the accuracy and efficiency of edge detection and provides reliable input data for the subsequent circle detection stage. The steps involved in image edge preprocessing are given in Algorithm 1.
**Algorithm 1: Image Edge Preprocessing****Input:** The image with a circle outline, the Gaussian kernel size $k_{s}$, the number of retained edges s, threshold value 1 is $th_{1}$ and threshold value 2 is $th_{2}$ in Canny edge detection algorithm.**Output:** Edge pixels under retention.1: Initialize $k_{s}$ = 9, s  = 6, $th_{1}$ = 200, $th_{2}$ = 255. 2: Calculate the $C_{m}$ by Canny edge detection algorithm and Formula (1). 3: Calculate the $C_{s}^{0}$ with (2). 4: Calculate the $C_{p^{s}}$ with (3).

### 3.2. Selection of Fitting Sample Points

The DBSCAN clustering algorithm separates the main contour sample points and interfering points. The algorithm clusters edges of arbitrary shapes and splits complex and irregularly shaped edges well using two parameters: the neighborhood radius and the minimum number of sample points within the circle determined by this neighborhood radius. 

**Figure 4:**
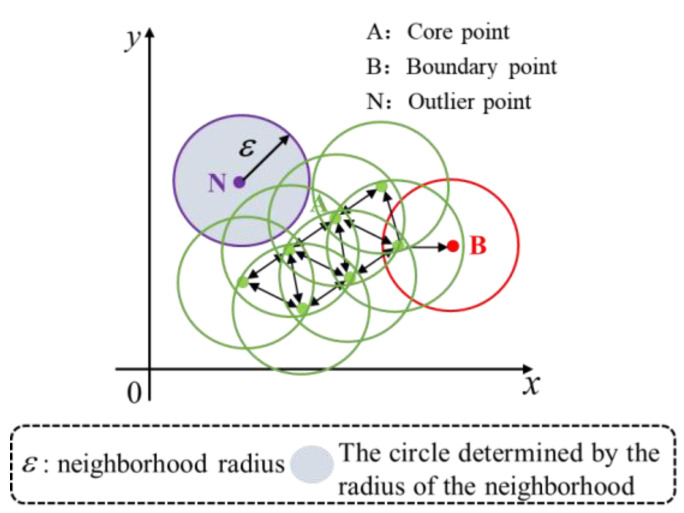
Clustering process of DBSCAN algorithm.

The DBSCAN algorithm clusters sample points into different classes based on the size of their neighborhood density. The clustering principle is shown in Figure 4. Formula (5) is used to classify the sample points. If the number of sample points in the neighborhood of sample point A is greater than or equal to the minimum number of sample points, the sample point A is classified as a core point. If the number of the sample points in B’s neighborhood are less than the minimum number of sample points, point B is classified as a boundary point. If the number of sample points in N’s neighborhood is 0, point N is classified as an outlier point.

(5) $$\left\{\begin{array}{c} (x_{c},y_{c})\in C_{A},n_{\varepsilon }\geq n_{\min pts}\\ (x_{c},y_{c})\in C_{B},1<n_{\varepsilon }<n_{\min pts}\\ (x_{c},y_{c})\in C_{N},n_{\varepsilon }=1 \end{array}\right.$$

where $\left(x_{c},y_{c}\right)$ represent the coordinates of the sample point, $C_{A}$ denotes the set formed by the core point A, $C_{B}$ represents the set created by the boundary point B, and $C_{N}$ indicates the set created by the outlier point N. $n_{\varepsilon }$ indicates the number of sample points in a circle centered at $\left(x_{c},y_{c}\right)$ with a radius of ε. Please note that after the screening of the fitted samples, the output is the set $C_{A}$.

If the sample point is marked as the core point, the above clustering process will be repeated for the sample points in the neighborhood until all sample points are marked.

**Figure 5:**
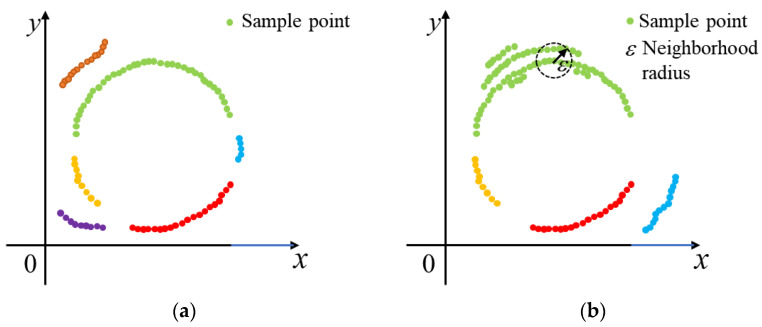
DBSCAN clustering results: (**a**) main contour; (**b**) main contour and outer interfering points. Note: Different-colored sample points in the figure represent different clustering results.

Two results occur after image preprocessing: the first is that the longest edge in the image includes only the main contour, as shown in Figure 5a; the other is that it contains both the main contour and the outer interfering points, as shown in Figure 5b. If clustering results with fewer sample points selected for fitting, they may suffer from image interfering points or broken edges. Therefore, the algorithm proposed in this paper retains the class with the most sample points. However, it is possible that interfering points that are close to the main contour are incorrectly clustered into fitted sample points due to unreasonable values of ε and $n_{\min pts}$. The algorithm specifically related to the selection of the fitted sample points is shown in Algorithm 2.
**Algorithm 2: Select the Fitting Sample Points****Input:** The $C_{p^{s}}$ in Algorithm 1, the radius of the neighborhood ε and the minimum number of points in the neighborhood $n_{\min pts}$ in the DBSCAN algorithm.**Output:** The edge set $C_{A}$ with the most sample points.1: Initialize ε = 5, $n_{\min pts}$ = 3.2: According to Section 3.2, clustering the sample points in $C_{p^{s}}$. 3: Calculate the number of sample points in each cluster and retain the class with the most sample points.

### 3.3. Candidate Circle Iteration and Ideal Circle Determination 

**Figure 6:**
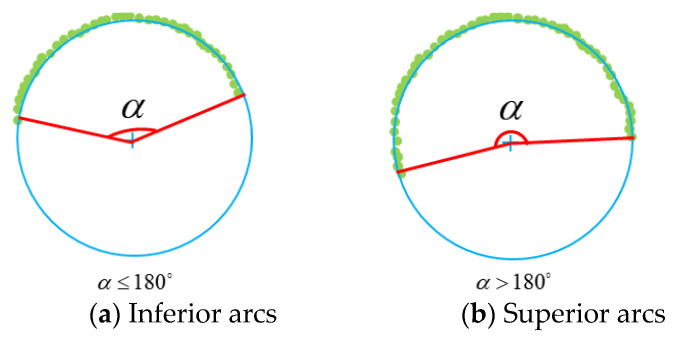
Inferior arcs and superior arcs.

A set of sample points with a main contour is obtained in Section 3.2, and the sample points show certain distributional features.

(1). Sample points on the main contour are connected into superior or inferior arcs. An arc with a central angle of less than or equal to 180° is called inferior arc, as shown in Figure 6a; an arc with a central angle of larger than 180° is called superior arc, as shown in Figure 6b.
(2). Besides the sample points of the main contour, a few interfering points are distributed on the outer side of the main contour. Fitting candidate and ideal circles in such cases is studied in this section.

Based on the comparison of the RHT algorithm, RANSAC algorithm, and LSC algorithm in the literature review, the LSC algorithm has a better fitting circle effect. To improve the accuracy and efficiency of single-circle detection, the algorithm uses the residual sum of squares to fit the circles.

**Figure 7:**
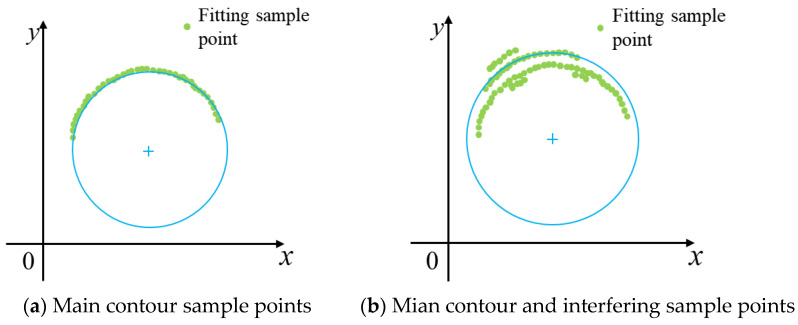
Conventional least squares fitting circle method.

**Figure 8:**
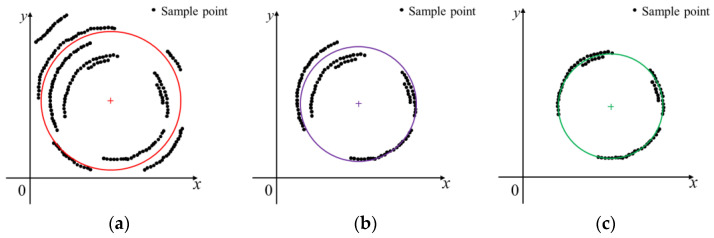
Principle of the method for removing the fitted failure points one by one based on the least squares method: (**a**) fitting of the first candidate circle; (**b**) fitting of the second candidate circle; (**c**) fitting of the third candidate circle.

It can be seen from Figure 7a, when only the main contour sample points exist in the fitting sample, the fitting results are better. However, the traditional least squares method is more sensitive to the interfering points, leading to errors in the results of the circle fitting, as shown in Figure 7b. To obtain the desired circle fitting results, this section proposes a method to remove the fitted failure points one by one based on the least squares method. This method introduces two parameters: the maximum number of iterations allowed and the critical residual sum of squares, based on the following principle (depicted in Figure 8).

As shown in Figure 8 (take the example of three iterations), the upper left interfering points is biased relative to the other interfering points. The first candidate circle is biased to the top left, sensitive to the interfering points, and has a large residual sum of squares. Interfering points outside the candidate circle are removed by comparing the distance from the sample points to the circle’s center with the radius. This sample point is kept if the distance is less than the radius. On the contrary, this sample point is deleted, as depicted in Figure 8a. Compared with Figure 8a, the distribution of sample points in Figure 8b are relatively uniform. As shown in Figure 8c, by removing the interfering points outside the candidate circle and fitting a third candidate circle, the fitted candidate circle gradually converges to the real-world circle. The specific iterative process is as follows:

(6) $$k\in \left[1,K\right],\;k\in Z$$

For each iteration *k*,

(7) $$Q_{k}=\sum_{i=0}^{n_{ip}}{\left[(x_{i}-a_{k}^{*}{)}^{2}+(y_{i}-b_{k}^{*}{)}^{2}-r_{k}^{*2}\right]}^{2}$$

where k represents the current number of iterations and K represents the maximum number of iterations allowed; $Q_{k}$ indicates the residual sum of squares of the *k*th iteration; $\left(a_{k}^{*},b_{k}^{*}\right)$, $r_{k}^{*}$ indicate the center coordinate and radius of the *k*th iteration, respectively; and $n_{ip}$ denotes the number of sample points used for the iteration.

The DBLSKCF algorithm obtains the optimal single-circle parameters by minimizing the residual sum of squares. Formula (6) is used to set a range of values for the number of iterations. The residual sum of squares for each fit is calculated by Formula (7). From Formula (7), it can be seen that $Q_{k}$ is a function of $a_{k}^{*}$, $b_{k}^{*}$, $r_{k}^{*}$. We can obtain the values of $a_{k}^{*}$, $b_{k}^{*}$, $r_{k}^{*}$ when $Q_{k}$ is minimized by Formula (8):

(8) $$\left\{\begin{array}{c} \frac{\partial Q_{k}}{\partial r_{k}^{*}}=0\\ \frac{\partial Q_{k}}{\partial a_{k}^{*}}=0\\ \frac{\partial Q_{k}}{\partial b_{k}^{*}}=0 \end{array}\right.$$

(9) $$r_{ki}={\left[(x_{i}-a_{k}^{*}{)}^{2}+(y_{i}-b_{k}^{*}{)}^{2}\right]}^{\frac{1}{2}},if\;Q_{k}\geq Q^{*}\;or\;k\leq K$$

where $Q^{*}$ denotes the critical residual sum of squares. $\left(x_{i},y_{i}\right)$ represent the coordinates of the sample points. $r_{ki}$ denotes the distance from the sample points to the center of the circle at the *k*th iteration. Please note that after the candidate-circle iteration and ideal-circle determination, the center coordinates and radius of all candidate circles are obtained. 

If the number of iterations is less than the maximum number of iterations allowed, or the residual sum of squares is greater than the critical residual sum of squares, then calculate the distance from each sample point to the center of the fitted circle using Formula (9). If the distance is less than the radius of the fitted candidate circle, the sample points are retained for the next fitting of the candidate circle. Instead, the sample points outside the fitted candidate circle are deleted. If K and $Q^{*}$ do not satisfy Formula (9), the iteration will be stopped, and the center coordinates and radius of the candidate circle are outputted. The corresponding algorithm for obtaining candidate and ideal circles are shown in Algorithm 3.

K and $Q^{*}$ are introduced to reduce the frequency with which underfitting or overfitting occurs. However, the numerical settings of the two parameters may lead to underfitting or overfitting. Under the joint action of the two parameters, ideal circle fitting results are shown in Table 1 below:

From Table 1, it can be seen that different values affect the ideal circle to various degrees; and therefore, the optimal value of the parameter needs to be determined. In this paper, twenty-six complex images of different types are randomly selected, including dials, wheels, traffic signs, etc., and the plot of the residual sum of squares versus the number of iterations determines the optimal combination.

**Figure 9:**
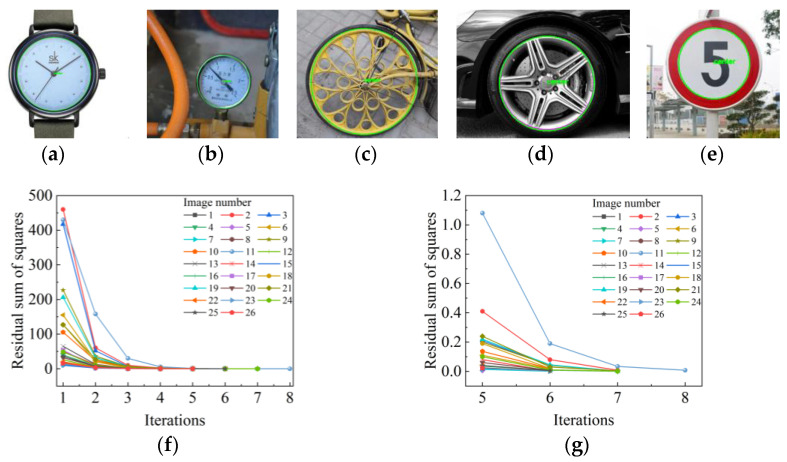
Determination of the optimal critical residual sum of squares and the maximum number of iterations allowed; (**a**–**e**) denote the detection results of circles in different complex scenarios; (**f**) shows the plot of the residual sum of squares versus the number of iterations; (**g**) represents the localized zoomed-in view after four iterations in (**f**).

Theoretically, the result of circle fitting is the best when $Q_{k}$ is close to 0. To prevent overfitting, the value of $Q_{k}$ is set to 0.0005, as well as the value of K to 100. According to Figure 9f, it can be seen that the residual sum of squares in different types of complex images has an identical trend of change. Due to the irregular distribution of the interfering points, the residual sum of squares for the first iteration is large, and the second iteration has a significant decrease, with large changes in the center coordinates and radius of the circle. After four iterations, the residual sum of squares decreases to a relatively stable value. As shown in Figure 9g, only a few images are subjected to the seventh iteration, and the slopes on both sides changed less before and after the sixth iteration. With the increase in the number of iterations during the subsequent iterations, the residual sum of squares ceases to change or decline in a small range. Therefore, we set the maximum number of iterations allowed in the DBLSKCF algorithm to six. The minimum value of the residual sum of squares in the sixth iteration is 0.003, and it makes it most reasonable to set it to 0.003 to make more images with only six iterations in single-circle detection. It balances ideal circle detection accuracy and efficiency with and together.
**Algorithm 3: Fit Candidate Circles and Determine Ideal Circles****Input:** The edge set $C_{A}$ in Algorithm 2, the maximum number of iterations allowed K, the critical residual sum of squares $Q^{*}$, the iteration number k.**Output:** Center ($a_{k}^{*}$,$b_{k}^{*}$) and radius $r_{k}^{*}$ of the candidate circle.1: Initialize K = 6, $Q^{*}$ = 0.003, *k* = 1.2: Calculate the $Q_{k}$, ($a_{k}^{*}$,$b_{k}^{*}$) and $r_{k}^{*}$ with (7) and (8). 3: **while** k≤K *or* $Q_{k}\geq Q^{*}$ **do** 4:   Calculate the $r_{ki}$ with (9). 5:   **if** $r_{ki}$ ≤ $r_{k}^{*}$ **then**
6:     Save ($x_{i}$,$y_{i}$) 7:   **else** 8:     Delete ($x_{i}$,$y_{i}$) 9:   **end if**
10:  Update k=k+1 11: **end while**

### 3.4. Improvement of Output Circle Detection Accuracy

**Figure 10:**
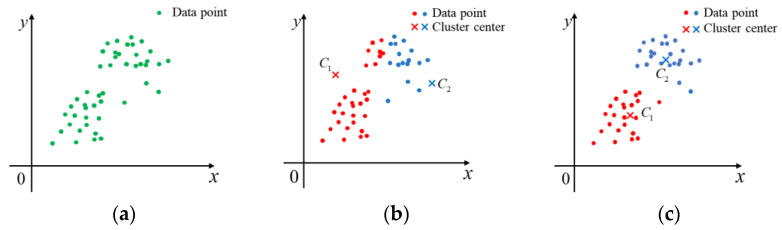
Schematic diagram of clustering process of K-means algorithm. (**a**) Original data points. (**b**) Start of clustering. (**c**) Clustering result.

Section 3.3 determines the ideal circle by $Q^{*}$ and K, reducing the frequency of occurrence of overfitting and underfitting. Error exists between the ideal circle and real-world circle due to various interfering points. To improve the accuracy of output circle detection, this section adopts the K-means clustering algorithm in machine learning to cluster the center coordinates and radius of all candidate circles to achieve the purpose of error compensation for output circle parameters. The clustering process of the K-means clustering algorithm is illustrated in Figure 10.

According to Figure 10, the data points are clustered into two clusters, and the clustering center is updated by calculating the distance from the data points to the clustering center. The distance is calculated using Formula (10), and the data points are assigned to the cluster with the closest distance.

(10) $$D_{j}=\sum_{i=1}^{N_{j}}{\left\|X_{i}-B_{j}\right\|}^{2},X_{i}\in S_{j},j\in n_{k}$$

where $S_{j}$ denotes the *j*th cluster and $B_{j}$ denotes the clustering center; $N_{j}$ indicates the number of data contained in the *j*th cluster; $n_{k}$ represents the number of clusters; $X_{i}$ denotes the data point in $S_{j}$; and $D_{j}$ denotes the distance from the data point to the corresponding clustering center.

From the clustering principle, it is necessary to minimize the distance from the data points in each cluster to the corresponding cluster center. $B_{j}$ is determined by taking partial derivatives of $D_{j}$ to $B_{j}$. As shown in Formulas (11) and (12):

(11) $$\frac{\partial }{\partial B_{j}}\sum_{i=1}^{N_{j}}{\left\|X_{i}-B\right\|}^{2}=0$$

(12) $$B_{j}=\frac{1}{N_{j}}\sum_{i=1}^{N_{j}}X_{i}$$

The final clustering result is obtained by a continuous iteration of Formulas (11) and (12). In Section 3.3, we can determine that K is 6 and $Q^{*}$ is 0.003. However, the final number of iterations may be less than 6. Therefore, the algorithm is discussed in terms of categorization based on the number of data points in the cluster results:

(1) Different numbers of data points in the two clustering results.

When the numbers of data points in the two clustering results differ, the algorithm proposed choose the cluster with more data points as the target cluster. The reason is as follows: the K-means clustering algorithm clusters data points based on their distance from the clustering center. The fitting results for the first few iterations vary widely, and the clustering algorithm clusters these data points into one cluster. In the later iterations, the fitting results change stably. The clustering algorithm will cluster these data points into one cluster, and clustering centers are close to data points. Therefore, the target cluster can be obtained by filtering the number of data points. Clustering results with many data points are obtained through Formula (13). Calculate the mean value of the circle parameter of the cluster using Formula (14), which is the result of the error compensation for the output circle, as follows:

(13) $$C=f(C_{1},C_{2})$$

(14) $$x=\frac{1}{n_{c}}\sum_{k=0}^{n_{c}}a_{k}^{*},y=\frac{1}{n_{c}}\sum_{k=0}^{n_{c}}b_{k}^{*},r=\frac{1}{n_{c}}\sum_{k=0}^{n_{c}}r_{k}^{*}$$

where $C_{1}$, $C_{2}$ indicate the set of data points in the results of the two clusters, respectively. f(•) is a function retaining the set with the most elements. C denotes the set of clustering results with more data points. $n_{c}$ represents the number of data points in set C. (x,y), r are the center coordinates and radius of the output circle, respectively.

(2) Same number of data points in two clustering results.

In this case, the algorithm selects clustering results based on the mean of the candidate circle radius. The reason is shown as follows: The fitting results for the first few iterations vary widely, and the clustering algorithm clusters these data points into one cluster. In the later iterations, the fitting results change stably. The mean of all candidate circles radius lie between the radius corresponding to the center of these two clusters. Formulas (15) and (16) are used to calculate the radius mean of the candidate circles in the two clustering results, respectively. Formula (17) are used to calculate the radius mean of all candidate circles. The target cluster is the cluster corresponding to the clustering result more minor than the mean by Formulas (18) and (19).

(15) $$r_{1}=\frac{1}{n_{1}}\sum_{k=0}^{n_{1}}r_{k}^{*}$$

(16) $$r_{2}=\frac{1}{n_{2}}\sum_{k=0}^{n_{2}}r_{k}^{*}$$

(17) $$r_{mean}=\frac{1}{n_{1}+n_{2}}\sum_{k=0}^{n_{1}+n_{2}}r_{k}^{*}$$

(18) $$R=\min\{r_{1},r_{2}\}$$

(19) $$x=\frac{1}{n_{R}}\sum_{k=0}^{n_{R}}a_{k}^{*},y=\frac{1}{n_{R}}\sum_{k=0}^{n_{R}}b_{k}^{*},\;r=R$$

where $r_{1}$ denotes the mean of the radius of the candidate circles in set $C_{1}$, $r_{2}$ denotes the mean of the radius of the candidate circles in set $C_{2}$,and $r_{mean}$ denotes the mean of the radius of all candidate circles. *R* represents the minimum value in $r_{1}$, $r_{2}$. $n_{1}$ and $n_{2}$ denote the number of data points in the two clustering results, respectively, and $n_{R}$ denotes the number of data points in the target cluster. Please note that the center coordinates and radius of the output circle are obtained after the improvement of output circle detection accuracy.

The corresponding algorithm to obtain a high precision output circle is proposed. Please see Algorithm 4. In this paper, the algorithm that does not use K-means clustering is called the DBLSCF algorithm. The method will be verified via specific experiments in Section 4 to improve the accuracy of output circle.
**Algorithm 4:** Improve the Output Circle’s Detection Accuracy**Input:** The k-means algorithm clustering number $n_{k}$, center ($a_{k}^{*}$,$b_{k}^{*}$) and radius $r_{k}^{*}$ of the candidate circle in Algorithm 3.**Output:** Center coordinates (x,y) and radius r of the output circle.1: Initialize $n_{k}$ *=* 2.2: According to the method in Section 3.4, the center coordinates are clustered into$\;C_{1}$ and$\;C_{2}$, respectively. 3: **if**
*num*($C_{1}$) *is not equal to num*($C_{2}$) **then** 4:   Calculate C with (13). 5:   Calculate (x,y) and r with (14). 6: **else** 7:   Calculate $r_{1}$, $r_{2}$ and $r_{mean}$ with (15), (16) and (17), respectively. 8:   **if** $r_{1}$< $r_{mean}$ **then** 9:     (x,y), r *= mean*(($a_{k}^{*}$,$b_{k}^{*}$),$r_{k}^{*}$) ($k\in (0,num(C_{1}))$ ) 10:  **if** $r_{2}$< $r_{mean}$ **then** 11:    (x,y), r *= mean*(($a_{k}^{*}$,$b_{k}^{*}$),$r_{k}^{*}$) ($k\in (0,num(C_{2}))$ ) 12:  **end if**
13: **end if**

## 4. Experiments and Results

This section compares the DBLSKCF algorithm with the RANSAC, RHT, and DBLSCF algorithms regarding detection accuracy, efficiency, and stability. Two groups of experiments are launched. The first experiments in Section 4.1 are conducted under laboratory conditions, using images captured with various lighting intensities. The experiments are designed to evaluate the stability of the four algorithms under different lighting conditions. Section 4.2 aims to verify the accuracy and efficiency of the DBLSKCF algorithm by the GH dataset [23]. The comparative experimental setup is as follows:

(1). All experiments are carried out using the same computer. The computer parameters are shown in Table 2.
(2). To comparatively validate the detection speed, the four algorithms are terminated as soon as a circle was detected in the image.

### 4.1. Comparison of Stability of Circle Detection

**Figure 11:**
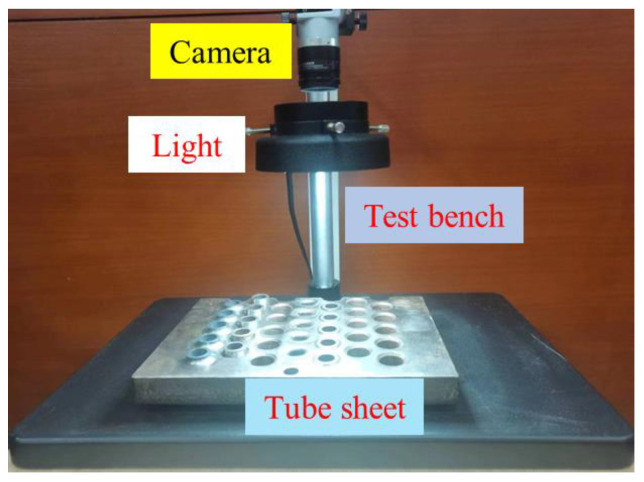
Experimental platform.

We use a mean light intensity of 650 lx and a standard deviation of 50 lx to simulate variations in light intensity and randomly select twenty-four datasets. The stability of the four algorithms in practical applications is evaluated by comparing the detection results under various light intensity. The edges detected in the experiment are selected to be the inner diameter edges of the steel pipe, accompanied by rust, scratches, and strong reflectivity on the end face. The experimental platform consists of an industrial camera, a white ring light source, and a tube sheet. The experimental platform is shown in Figure 11.

In the experiment, the center coordinates and the radius change are used as stability measures. Under various light intensities, if the center coordinates and radius only change in a small range, it indicates that the algorithm has high stability and can effectively resist external interference. On the contrary, the algorithm is less stable and less resistant to external interference.

**Figure 12:**
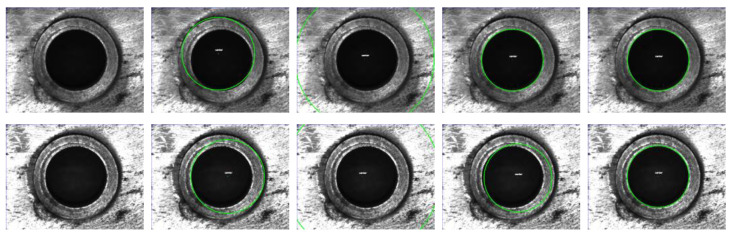

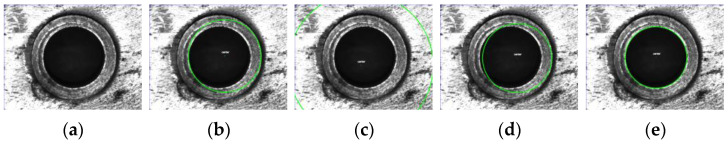
Circle detection results under various light intensities. From top to bottom: the light intensity is 598 lx, 645 lx, and 686 lx, respectively. (**a**) Original image; (**b**–**e**) denote the single-circle detection results of the RANSAC, RHT, DBLSCF, and DBLSKCF algorithms, respectively.

**Figure 13:**
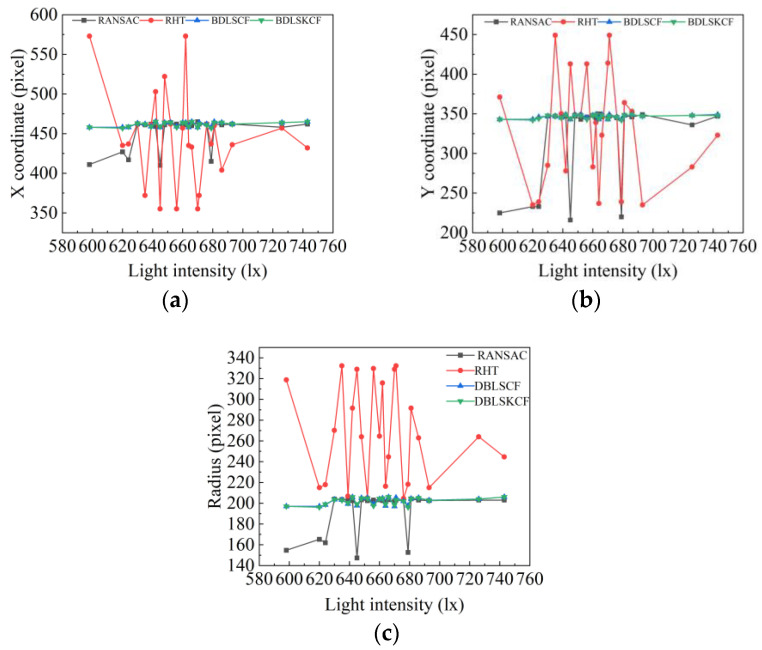
Detection results of four algorithms under various light intensities. (**a**) X-coordinate change. (**b**) Y-coordinate change. (**c**) Radius change.

From Figure 12, for the RHT, RANSAC, and DBLSCF algorithms, the algorithm’s circle detection results under different light intensities are highly differentiated, and the detection results of the DBLSKCF algorithm maintain almost the same. All results are shown in Figure 13. With the increase in light intensity, the circle detection results of the RHT algorithm change unstably. In contrast, the circle detection results of the RANSAC algorithm tend to be stable. In addition, the circle detection results of the DBLSKCF and DBLSCF algorithms vary within a small range. We use the standard deviation from Table 3 to measure the algorithm’s stability better. A significant standard deviation indicates greater variability in the circle detection results, more excellent dispersion, and lower stability. Both in terms of coordinates of x and y, and radius, the detection results of the RHT algorithm have the more significant standard deviation and the worst stability. The detection results of the RANSAC algorithm show substantial variations in the relatively weak phase of light intensity and gradually stabilize with light intensity enhancement. The detection results of the DBLSCF and DBLSKCF algorithms change almost synchronously. But by calculating the standard deviation, it is found that the DBLSKCF algorithm has better stability. Therefore, the DBLSKCF algorithm has better stability and resistance to external interference.

### 4.2. Validation of Algorithm Detection Accuracy and Efficiency

To verify the accuracy and efficiency of the algorithm in this paper, we validate it with the GH dataset. The images in the GH dataset cover a variety of scenes and backgrounds, including indoor and outdoor environments, different lighting conditions, and different levels of an object and background clutter. The GH dataset is therefore well suited for testing and training the robustness and generalization of circle detection algorithms. Forty-eight images with a single circle are selected from the GH dataset, each labeled with the corresponding circle parameter.

**Figure 14:**
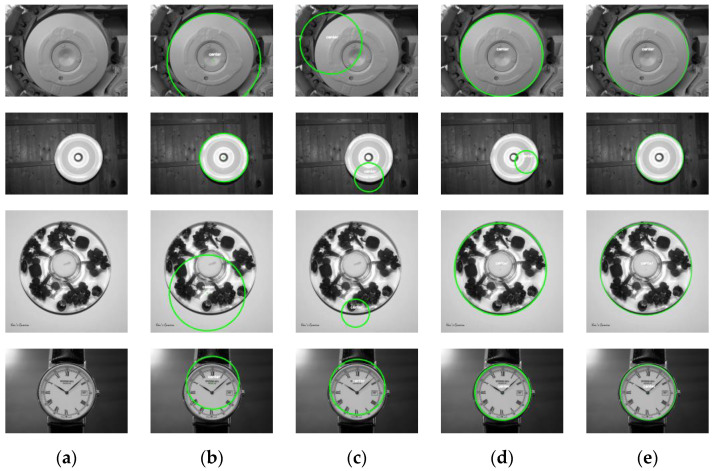
Circle detection results of four algorithms. (**a**) Original image. (**b**) RANSAC. (**c**) RHT. (**d**) DBLSCF. (**e**) DBLSKCF.

As seen from Figure 14, the single-circle detection results of the RANSAC, RHT, and DBLSCF algorithms show varying degrees of fitting error, and only the results of the DBLSKCF algorithm are closest to the real-world circle parameters. The four algorithms’ circle detection results and running times are specified below.

**Figure 15:**
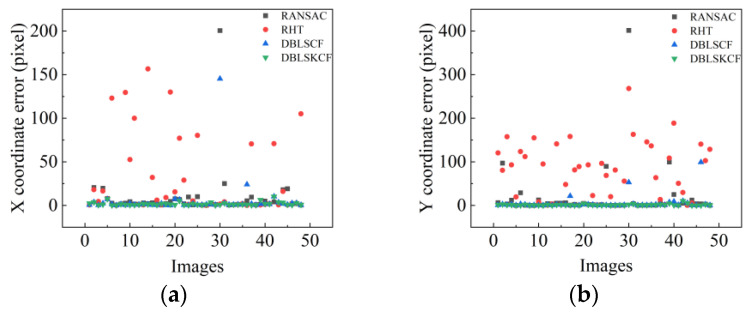

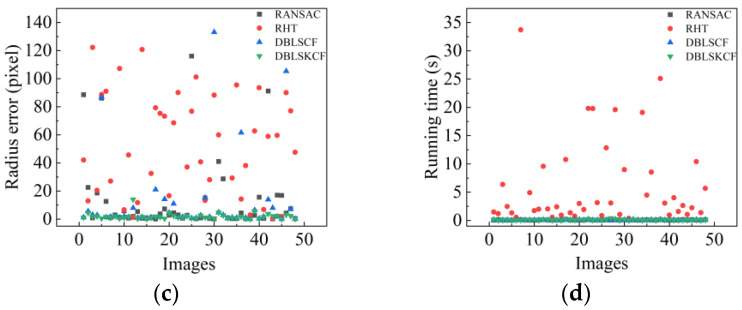
Detection results of different images for four algorithms. (**a**) X-coordinate error. (**b**) Y-coordinate error. (**c**) Radius error. (**d**) Running time. Note: Where the RHT algorithm has circle detection failures for a few images in the dataset, these are represented by breakpoints.

After analyzing Figure 15 and Table 4, the single-circle detection results of the RHT algorithm have high error and low efficiency. Please find Table A1, Table A2, Table A3 and Table A4 in the Appendix A for the data results of the experiments, respectively. The RANSAC algorithm performs better in efficiency, but has a higher error than the DBLSCF and DBLSKCF algorithms because, in the RHT and RANSAC algorithms, the sample points for fitting the circle are chosen randomly, resulting in the selected sample points not being points on the main contour. Since the DBLSCF algorithm outputs an ideal circle as an output circle, the ideal circle may be affected by interfering points, resulting in a significant deviation of the circle parameters. Thus, the circle detection accuracy of the DBLSKCF algorithm is 3–5 times higher than that of the DBLSCF algorithm. The experimental results also justify the K-means clustering algorithm to improve the accuracy of circle detection. Considering the circle detection accuracy and efficiency, the DBLSKCF algorithm is significantly better than the other compared algorithms.

## 5. Conclusions

This paper proposes a single-circle detection algorithm, i.e., the DBLSKCF algorithm, that combines Canny edge detection, two clustering algorithms, and improved least squares method. The proposed algorithm has proven to be an excellent solution to single-circle detection in complex images. Compared with RHT, RANSAC, and DBLSCF, DBLSKCF demonstrates clear advantages in detection accuracy and stability. The highlights (and also the core steps) of the detection methods are summarized below:

1. Image edge preprocessing removes as many interfering points as possible while retaining the main contour edge information.
2. The DBSCAN algorithm is utilized to cluster the main contours and interfering points into different clusters, from which the cluster with more sample points is extracted as the fitting samples of the candidate circles.
3. An improved least square fitting of the circle with the residual sum of squares is raised. Removing the fitting failure points one by one makes the circle fitting result gradually closer to the real-world circle.
4. The K-means clustering algorithm is implemented to cluster the center coordinates and radius of all candidate circles to improve the accuracy of output circle detection.

Performance of the DBLSKCF algorithm:

(1). Stability: The standard deviation of the X-coordinate, Y-coordinate, and radius detection results are 2.7 pixels, 2.3 pixels, and 3.27 pixels, respectively.
(2). Detection accuracy: The average errors of X-coordinate, Y-coordinate, and radius detection are 1.8 pixels, 1.4 pixels, and 1.9 pixels, respectively.
(3). Running time: The average running time is 0.1 s.

By comparing the detection performance with other algorithms, the proposed DBLSKCF algorithm outperforms in detection accuracy and stability. 

Future work will be carried out in two main perspectives:

(1). Adaptively determining the neighborhood radius and the minimum number of sample points within the neighborhood radius in the DBSCAN clustering algorithm.
(2). An improvement of the proposed algorithm to enable multi-circle detection.

## Figures and Tables

**Table 1:** Effect of K and $Q^{*}$ on the ideal circle fitting result.

*K*	Q*	Ideal Circle
Large	Large	underfitting
Large	Small	overfitting
Small	Large	underfitting
Small	Small	overfitting

**Table 2:** Specific parameters of the operating computer.

Development Environment	Internal Storage	Executive System	Development Tool
CPU:AMD Ryzen 7 6800HS Creator Edition 3.20 GHz	16 G	Windows 11	Python 3.6

**Table 3:** Standard deviation of circle detection results for four algorithms.

Algorithm	RANSAC	RHT	DBLSCF	DBLSKCF
X coordinate (pixel)	18.7	58.1	2.8	2.7
Y coordinate (pixel)	49.2	66.6	2.4	2.3
Radius (pixel)	19.2	46.3	3.37	3.27

**Table 4:** Comparison of circle detection mean value.

Algorithm	RANSAC	RHT	DBLSCF	DBLSKCF
X-coordinate error (pixel)	8.9	28.4	5.3	1.8
Y-coordinate error (pixel)	36.5	77.2	5.2	1.4
Radius error (pixel)	13.3	51	11.4	1.9
Running time (s)	0.08	6	0.07	0.1

## Data Availability

Data available on request from the authors.

## Appendix A

### Comparison Result of the DBLSKCF Algorithm with the Other Three Algorithms

**Table A1:** X-coordinate error (pixel).

Image Number	RANSAC	RHT	DBLSCF	DBLSKCF
1	1.43	0.5	0.51	2.38
2	20.5	18	3.74	3.73
3	2.26	4.5	0.32	0.41
4	19.71	16.5	2.32	1.09
5	8	7.5	7.15	7.41
6	2.5	123	1.31	0.08
7	0.46	0	0.05	0.18
8	1.67	-	1.49	1.49
9	2.5	129.5	0.36	0.65
10	4.26	52.5	2.19	0.38
11	1.5	100	0.71	0.38
12	1.24	0.88	0.89	0.9
13	2.81	0.49	1.55	1.75
14	2	156.5	1.14	1.15
15	3	32	0.39	1.43
16	5	6	0.3	0.37
17	0.39	0.5	2.19	2.04
18	2.02	9	0.21	1.85
19	4.5	130	0.07	1.14
20	7.48	15.5	7.384	0.7
21	6.83	77	6.7	5.31
22	1.87	29	0.48	0.59
23	9.65	1	0.35	0.35
24	0.5	5	1.92	2.04
25	10	80.29	0.75	0.84
26	0.36	0.5	0.57	0.57
27	0.27	0	1.09	1.09
28	1.37	2.06	1.34	3.5
29	1.01	0.5	0.55	0.17
30	200.5	1.5	145.34	0.92
31	25	4	2.55	2.53
32	0.48	-	0.72	0.68
33	0.79	-	0.87	1
34	1.1	1	1.04	1.03
35	1.21	2	1.21	1.1
36	5.2	0	24.09	0.83
37	9.5	70.5	1.9	0.25
38	0.8	0.18	0.48	0.45
39	1.25	0.5	1.52	6.44
40	5	1	1.55	1.76
41	1.73	2	1.58	1.69
42	4	70.75	10.25	10.23
43	2.14	0.7	3.43	3.58
44	17.94	16	1.88	3.21
45	19.08	0.46	0.8	0.8
46	1.81	2	2.54	0.39
47	2.021	2.5	2.72	2.71
48	0.37	105	0.48	0.69

Note: The “-” in the table means that the algorithm did not detect the circle in the corresponding image.

**Table A2:** Y-coordinate error (pixel).

Image Number	RANSAC	RHT	DBLSCF	DBLSKCF
1	6.27	120.5	0.61	1.48
2	97	80.5	2.66	2.68
3	3.13	157.5	1.25	1.28
4	11.87	93	2.15	1.05
5	0	19.5	0.2	0.12
6	29	123.5	3.18	0.35
7	0.46	112	1.86	1.88
8	0.1	-	0.09	0.11
9	0	155	0.53	0.53
10	12.5	8	0.2	0.23
11	0.5	95	0.12	0.1
12	4.09	0.46	1.82	1.84
13	3.4	3.26	0.21	0.12
14	5	141	1.53	1.55
15	5	1.5	1.74	1.47
16	6	48	1.03	1
17	0.48	158	21.46	0.85
18	2.17	81.5	0.245	0.03
19	0.5	89	0.38	0.29
20	4.45	4	1.925	4.6
21	2.05	93	2.241	1.7
22	1.69	22.5	1.36	1.24
23	0.95	0.65	0.42	0.39
24	1.8	96.5	0.44	0.34
25	89.5	68.75	0.66	0.75
26	0.02	20	0.41	0.38
27	0.14	81	0.33	0.33
28	0.29	0.05	0.09	1.5
29	0.69	55.5	0.94	0.32
30	401.5	268	53	2.02
31	4	163	3.3	3.28
32	0.11	-	0.29	0.26
33	0.39	-	0.92	0.88
34	0.32	145.5	0.54	0.51
35	0.59	136.5	1.07	1.1
36	0.44	63.5	2.05	1.4
37	6	13.5	1.56	2.04
38	1.35	1.79	1.28	1.25
39	99.6	108.5	7.29	4.65
40	25	188.5	8.71	0.5
41	0.75	50.5	0.95	0.35
42	5	29.61	10.26	10.25
43	1.19	0.24	5.42	5.47
44	12.17	5.5	0.03	0.07
45	3.66	0.05	0.03	0.02
46	3.3	140.5	99.14	0.03
47	2.75	102.5	2.22	2.19
48	0.28	128.5	0.52	0.72

Note: The “-” in the table means that the algorithm did not detect the circle in the corresponding image.

**Table A3:** Radius error (pixel).

Image Number	RANSAC	RHT	DBLSCF	DBLSKCF
1	88.55	42	1.2	1.13
2	22.55	12.96	5.53	4.45
3	0.82	122.25	3.08	1.96
4	18.52	20.5	2.81	2
5	86.09	88.75	86.73	0.49
6	12.59	91	1.28	1.66
7	0.91	27	1.63	1
8	2.99	-	1.97	1.98
9	1.19	107.25	0.95	0.38
10	5.81	6.59	1.8	1
11	0.61	45.62	1.39	1.69
12	1.87	1.14	7.94	13.99
13	5.36	11.73	0.84	0.36
14	0.53	120.75	1.51	1.53
15	0.49	0.62	0.7	0.07
16	1	32.45	1.22	0.93
17	0.02	79.25	20.99	1.91
18	3.62	75.25	1.56	1.49
19	7.25	73.25	14.19	0.32
20	2.73	16.59	4.48	4.4
21	4.48	68.54	10.94	2.46
22	2.97	90.09	1.35	1.09
23	0.55	1.18	1.52	1.34
24	2.71	37	2.27	2.26
25	116.04	76.78	0.58	0.61
26	0.22	101.19	0.72	0.65
27	0.82	40.75	2.64	2.63
28	15.06	13.27	15.26	1.2
29	0.4	28	1.57	1
30	0.13	88.25	133.16	0.35
31	41	59.94	4.69	4.69
32	28.66	-	2.84	2.8
33	0.95	-	0.51	0.67
34	0.29	29.25	1	0.83
35	0.37	95.5	0.84	0.67
36	4.38	14.24	61.55	1.85
37	0.25	38.07	0.89	0.28
38	1.76	2.89	0	0.06
39	2.69	62.75	6.4	5.65
40	15.59	93.5	0.36	0.29
41	0.47	6.75	1.72	1.54
42	91.18	58.9	13.93	3.78
43	0.09	0.46	7.97	2.15
44	17.03	59.62	1.75	2.36
45	16.83	1.74	0.09	0.01
46	4.27	90.05	105.35	3.1
47	7.31	77	7.29	2.24
48	0.42	47.53	0.04	0.15

Note: The “-” in the table means that the algorithm did not detect the circle in the corresponding image.

**Table A4:** Running time (s).

Image Number	RANSAC	RHT	DBLSCF	DBLSKCF
1	0.06	1.49	0.099	0.177
2	0.139	1.196	0.026	0.033
3	0.059	6.37	0.05	0.059
4	0.061	2.48	0.058	0.104
5	0.051	1.34	0.024	0.025
6	0.164	0.55	0.033	0.061
7	0.123	33.72	0.067	0.079
8	0.079	-	0.063	0.122
9	0.062	4.91	0.067	0.099
10	0.058	1.73	0.057	0.09
11	0.056	1.99	0.035	0.038
12	0.129	9.58	0.113	0.233
13	0.067	2.03	0.07	0.122
14	0.053	0.559	0.028	0.036
15	0.068	2.407	0.066	0.096
16	0.057	0.92	0.026	0.029
17	0.223	10.77	0.1	0.234
18	0.059	1.35	0.071	0.109
19	0.06	0.739	0.055	0.066
20	0.069	2.99	0.134	0.177
21	0.062	1.931	0.068	0.076
22	0.143	19.81	0.206	0.263
23	0.053	19.8	0.046	0.062
24	0.065	3.14	0.069	0.121
25	0.057	0.85	0.038	0.083
26	0.067	12.84	0.083	0.208
27	0.05	3.078	0.031	0.419
28	0.063	19.59	0.177	0.243
29	0.053	1.05	0.028	0.033
30	0.101	8.98	0.082	0.161
31	0.054	0.387	0.02	0.029
32	0.076	-	0.164	0.168
33	0.059	-	0.059	0.114
34	0.052	19.086	0.033	0.041
35	0.119	4.48	0.042	0.054
36	0.059	8.55	0.13	0.174
37	0.043	0.123	0.017	0.024
38	0.17	25.1	0.146	0.198
39	0.06	3.054	0.066	0.096
40	0.06	0.94	0.047	0.064
41	0.052	4.02	0.066	0.08
42	0.058	1.57	0.046	0.063
43	0.155	2.65	0.051	0.062
44	0.057	1.05	0.043	0.059
45	0.133	2.24	0.035	0.041
46	0.061	10.4	0.124	0.114
47	0.055	1.37	0.037	0.046
48	0.129	5.68	0.122	0.102

Note: The “-” in the table means that the algorithm did not detect the circle in the corresponding image.
